# Postoperative Complications in Patients with Frailty Syndrome Undergoing Esophagectomy—A Systematic Review with Meta-Analysis

**DOI:** 10.3390/jcm15083040

**Published:** 2026-04-16

**Authors:** Anna Kamińska, Michał Bonczar, Dawid Plutecki, Patryk Ostrowski, Mateusz Koziej, Aleksander Konturek

**Affiliations:** 1 Doctoral School of Medical and Health Sciences, Jagiellonian University Medical College Krakow, University Hospital in Krakow, 31-530 Kraków, Poland; 2 Department of Anatomy, Jagiellonian University Medical College Cracow, 33-332 Kraków, Poland; 3 Youthoria, Youth Research Organization, 30-363 Kraków, Poland

**Keywords:** frailty syndrome, esophagectomy, esophageal cancer, surgery, mortality

## Abstract

**Introduction:** Frailty syndrome is an increasingly recognized condition that affects a considerable proportion of elderly patients, particularly those undergoing major surgeries. In this meta-analysis, we aimed to systematically review and pool data from cohort studies to assess the effect of frailty on the clinical outcomes of patients undergoing esophagectomy for esophageal cancer. **Methods:** Major online medical databases such as PubMed, Embase, Scopus, and Web of Science were searched to gather all studies on the clinical outcomes of patients with frailty syndrome who underwent esophagectomy due to esophageal cancer. The study included articles published up to March 2026. Finally, 15 articles matched the required criteria and were included in this meta-analysis. **Results:** The pooled odds ratio for surgery-related mortality in patients with frailty syndrome and esophageal cancer undergoing esophagectomy has been established at 4.03 (Lower Limit: 2.20; Upper Limit: 7.38; *p*-value < 0.05). The pooled odds ratio for surgery-related postoperative pneumonia in patients with frailty syndrome and esophageal cancer undergoing esophagectomy has been established at 1.86 (Lower Limit: 1.16; Upper Limit: 2.98; *p*-value < 0.05). The pooled odds ratio for surgery-related postoperative cardiac complications in patients with frailty syndrome and esophageal cancer undergoing esophagectomy has been established at 1.73 (Lower Limit: 1.54; Upper Limit: 1.94; *p*-value < 0.05). **Conclusions:** Frailty is a powerful predictor of mortality in patients undergoing esophagectomy, with frail individuals facing nearly four times higher odds of death. This underscores the urgent need to integrate frailty assessments into standard preoperative screening to enhance risk stratification and optimize perioperative decision-making. A multidisciplinary approach is essential to improving resilience, recovery, and long-term survival in frail esophageal cancer patients. Future large-scale prospective trials should focus on standardizing assessment tools and evaluating the lasting impact of tailored interventions to ultimately enhance patient outcomes.

## 1. Introduction

Frailty syndrome is an increasingly recognized condition that affects a considerable proportion of elderly patients, particularly those undergoing major surgeries, such as esophagectomy for esophageal cancer [1]. Frailty is a multidimensional condition characterized by decreased physiological reserves and increased vulnerability to stressors, resulting in a higher risk of adverse outcomes post-surgery [1,2,3,4]. Frailty reflects a decline in physiological reserve and multisystem dysfunction, leading to increased vulnerability to surgical stressors [5]. Reduced cardiopulmonary reserve, malnutrition, and impaired immune response in frail patients may compromise their ability to tolerate major surgery, contributing to higher rates of postoperative complications and mortality [6,7]. The burden of frailty is especially prominent in esophageal cancer, where the intensive nature of esophagectomy demands considerable physical and metabolic resilience from patients [4]. Esophageal cancer predominantly affects older adults, a population in which frailty is highly prevalent and often underdiagnosed. Esophageal cancer remains a significant global health burden, ranking as the 11th most common cancer with over 500,000 new cases and the seventh leading cause of cancer-related mortality worldwide [8,9]. Furthermore, esophagectomy, as the cornerstone of curative treatment, is associated with a high incidence of postoperative complications, affecting up to 76% of patients in contemporary cohorts [10]. As life expectancy increases and surgical indications expand to include older and more comorbid patients, the coexistence of frailty and esophageal cancer has become a more common clinical scenario. Importantly, frailty is distinct from chronological age and comorbidity burden, although it frequently coexists with both. While traditional preoperative risk assessment focuses on comorbid conditions and physiological parameters, increasing evidence suggests that frailty provides additional prognostic information and should be incorporated into preoperative risk stratification in older surgical patients [11,12,13].

The impact of frailty on surgical outcomes has been studied across various surgical procedures, showcasing an increased incidence of complications, prolonged hospital stays, and mortality among frail individuals [1,14,15,16,17,18,19,20,21]. In esophagectomy, where the procedure itself is complex and associated with high morbidity and mortality, frail patients are particularly susceptible to adverse events, including increased mortality, surgical site infections, postoperative leakage, postoperative pneumonia, and cardiac complications [1,22,23]. Understanding the influence of frailty on these outcomes is critical for improving preoperative assessment and tailoring postoperative care to minimize risks.

Despite growing interest in frailty assessment in surgical oncology, existing evidence regarding its impact on outcomes after esophagectomy remains limited. In this meta-analysis, we aimed to systematically review and pool data from various studies to assess the effect of frailty on the clinical outcomes of patients undergoing esophagectomy for esophageal cancer. By comparing frail and non-frail patients across mortality and major postoperative complications, we seek to demonstrate the risks associated with frailty, providing evidence to guide perioperative management strategies and enhance patient outcomes. A clearer understanding of the prognostic value of frailty may support the implementation of standardized frailty screening and facilitate more individualized perioperative decision-making in patients undergoing esophagectomy.

## 2. Materials and Methods

### 2.1. Search Strategy

Major online medical databases such as PubMed, Embase, Scopus, and Web of Science were searched to gather all studies on the clinical outcomes of patients with frailty syndrome who underwent esophagectomy due to esophageal cancer. The following search terms were used: ((esophageal cancer) OR (esophageal neoplasm*) OR (esophageal tumor*) OR (esophageal carcinoma)) AND ((frailty*) OR (frail*) OR (frailties) OR (fragility) OR (frailty syndrome) OR (Frail Elderly*)). The search terms were individually adapted to each database. No restrictions were imposed regarding date, article type, or text availability. Additionally, a supplementary search was performed through the references of the identified studies to ensure the thoroughness of the process. The study included articles published up to March 2026. The literature search was performed by two independent researchers (AKa and DP). During the study, the Preferred Reporting Items for Systematic Reviews and Meta-Analyses (PRISMA) guidelines were followed (Supplementary Material). This study was not prospectively registered in any database.

### 2.2. Eligibility Assessment and Data Extraction

**Figure 1:**
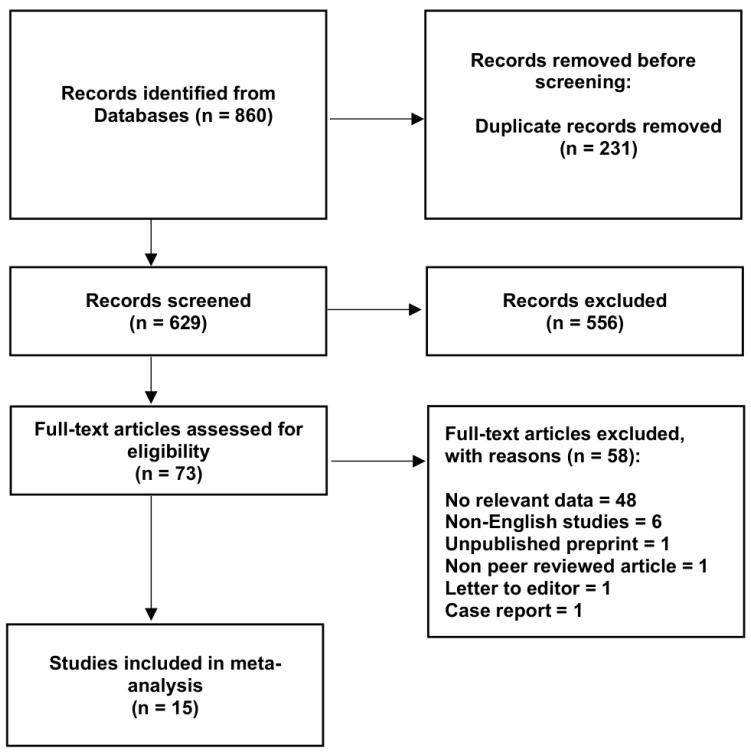
Flow diagram presenting the process of collecting data included in this meta-analysis.

Initially, a total of 860 studies were identified and evaluated by two independent researchers (AKa and DP). Out of those, 73 were qualified for a full-text evaluation. The following inclusion criteria were used: original articles with extractable numerical data that compared the clinical outcomes of patients with and without frailty syndrome after esophagectomy performed as part of the treatment of esophageal cancer. The exclusion criteria included conference reports, case reports, case series, reviews, letters to the editor, studies lacking relevant or compatible data, and non-English studies. Finally, 15 articles matched the required criteria and were included in this meta-analysis [1,7,22,24,25]. The overall process of collecting data is shown in Figure 1. Characteristics of all the studies included in this meta-analysis are presented in Table 1. Data from qualified studies were extracted by two independent reviewers (AKa and DP). Numerical data about the size of each cohort and the occurrence of any complications after the surgery in each group were gathered. Additional data regarding the patients’ age, sex, tumor stage, tumor location, BMI and comorbidities were also gathered. Any discrepancies between studies as identified by the two reviewers were resolved by contacting the authors of the original studies, wherever possible, or by consensus with a third researcher (MB).

### 2.3. Statistical Analysis

To perform the meta-analyses, STATISTICA version 13.1 software (StatSoft Inc., Tulsa, OK, USA) and Comprehensive Meta-analysis version 4.0 software (Biostat Inc., Englewood, NJ, USA) were used. A random-effects model was used in all analyses. The heterogeneity among the studies was evaluated using both the Chi-squared test and the I-squared statistic [35]. The I-squared statistic was interpreted as follows: 0–40% as “might not be important”; 30–60% as “may represent moderate heterogeneity; 50–90% as “may represent substantial heterogeneity”; and 75–100% as “may represent considerable heterogeneity” [35]. A *p*-value of less than 0.05 was considered to be statistically significant.

## 3. Results

Each qualified article was assessed using the Newcastle–Ottawa scale in order to prevent potential bias [36]. A star was not awarded when the study did not meet the predefined Newcastle–Ottawa scale criteria for a given domain, including insufficient methodological description, lack of representativeness, absence of appropriate control selection, inadequate adjustment for confounders, or incomplete or insufficient follow-up. The Newcastle–Ottawa scale assigns a maximum of nine stars to each study. Studies scoring 7–9 stars were considered high quality with low risk of bias, those scoring 5–6 stars were considered of moderate quality, and those scoring below 5 stars were classified as low quality with a high risk of bias. The results of the risk-of-bias assessment are presented in Table 2.

Comparison of the surgical-related clinical outcomes between patients with and without frailty syndrome undergoing esophagectomy due to esophageal cancer was assessed with respect to five main categories: (1) mortality; (2) surgical site infection; (3) postoperative leakage; (4) postoperative pneumonia; and (5) cardiac complications. Statistical results can be found in Table 3. Some of the studies included in this review were not included in all of the analyses due to the absence of sought data, lack of a precise stratification of outcomes between frail and non-frail patients or the presentation of data in a manner that precludes pooling. Nevertheless, the authors believe that the results of those studies are relevant in the context of this review and are presented further in a descriptive manner.

**Figure 2:**
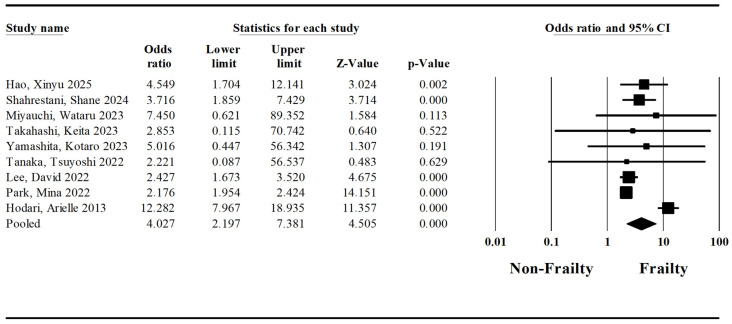
Forest plot regarding the impact of the frailty syndrome on the mortality of patients undergoing esophagectomy [1,7,22,24,25,26,31,33,34].

A pooled analysis regarding mortality was conducted based on nine studies by Hao et al. [26], Shahrestani et al. [1], Miyauchi et al. [22], Takahashi et al. [24], Yamashita et al. [31], Tanaka et al. [33], Lee et al. [7], Park et al. [34], and Hodari et al. [25]. The pooled odds ratio for surgery-related mortality in patients with frailty syndrome and esophageal cancer undergoing esophagectomy was established at 4.03 (Lower Limit: 2.20; Upper Limit: 7.38; *p*-value < 0.05) (Figure 2). The I-squared statistic is 87%, which indicates that 87% of the variance in observed effects reflects variance in true effects rather than sampling error.

**Figure 3:**
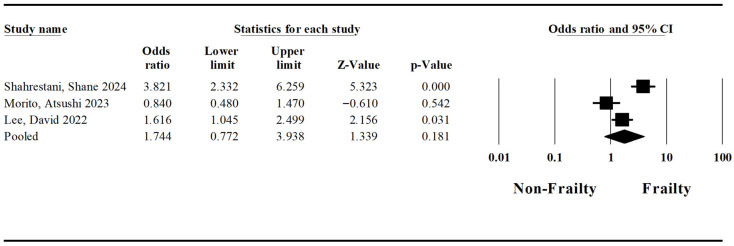
Forest plot regarding the impact of the frailty syndrome on infection of the surgical site in patients undergoing esophagectomy [1,7,23].

A pooled analysis regarding surgical site infection was conducted based on three studies by Shahrestani et al. [1], Morito et al. [23], and Lee et al. [7]. The pooled odds ratio for surgical site infection in patients with frailty syndrome and esophageal cancer undergoing esophagectomy was established at 1.74 (Lower Limit: 0.77; Upper Limit: 3.94; *p*-value: 0.18) (Figure 3). The I-squared statistic is 88%, which indicates that 88% of the variance in observed effects reflects variance in true effects rather than sampling error.

**Figure 4:**
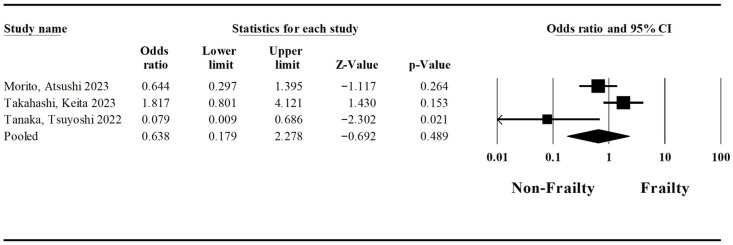
Forest plot regarding the impact of the frailty syndrome on postoperative leakage in patients undergoing esophagectomy [23,24,33].

A pooled analysis regarding postoperative leakage was conducted based on three studies by Morito et al. [23], Takahashi et al. [24], and Tanaka et al. [33]. The pooled odds ratio for postoperative leakage in patients with frailty syndrome and esophageal cancer undergoing esophagectomy was established at 0.64 (Lower Limit: 0.18; Upper Limit: 2.28; *p*-value: 0.49) (Figure 4). The I-squared statistic is 76%, which indicates that 76% of the variance in observed effects reflects variance in true effects rather than sampling error.

**Figure 5:**
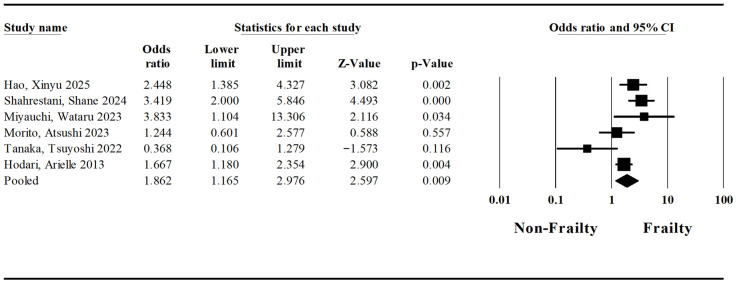
Forest plot regarding the impact of the frailty syndrome on postoperative pneumonia in patients undergoing esophagectomy [1,22,23,25,26,33].

A pooled analysis regarding postoperative pneumonia was conducted based on six studies by Hao et al. [26], Shahrestani et al. [1], Miyauchi et al. [22], Morito et al. [23], Tanaka et al. [33], and Hodari et al. [25]. The pooled odds ratio for surgery-related postoperative pneumonia in patients with frailty syndrome and esophageal cancer undergoing esophagectomy was established at 1.86 (Lower Limit: 1.16; Upper Limit: 2.98; *p*-value < 0.05) (Figure 5). The I-squared statistic is 67%, which indicates that 67% of the variance in observed effects reflects variance in true effects rather than sampling error.

**Figure 6:**
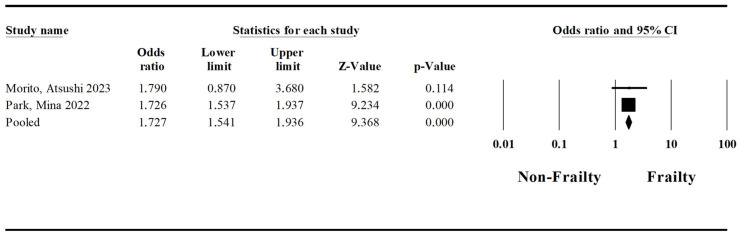
Forest plot regarding the impact of the frailty syndrome on cardiac complications in patients undergoing esophagectomy [23,34].

A pooled analysis regarding postoperative cardiac complications was conducted based on two studies by Morito et al. [23] and Park et al. [34]. The pooled odds ratio for surgery-related postoperative cardiac complications in patients with frailty syndrome and esophageal cancer undergoing esophagectomy was established at 1.73 (Lower Limit: 1.54; Upper Limit: 1.94; *p*-value < 0.05) (Figure 6). The I-squared statistic could not be calculated for this category, as a minimum of three studies is required to compute this measure. This highlights a gap in the literature and underscores the need for further research to provide sufficient data for analysis.

The following section summarizes the results of studies included in the systematic review but not incorporated into the quantitative synthesis due to the reasons mentioned above. The predictive value of frailty in esophagectomy outcomes was evaluated by Boerner et al. [28]. In a cohort of 447 patients, increasing frailty was associated with a higher risk of major complications (32%), readmissions (19%), and discharge to a facility (7.2%), although no significant association with 90-day mortality (4.9%) was found. The role of perioperative risk stratification tools was assessed by Chaochankit et al. [27]. High postoperative morbidity occurred in 48% of patients, with ASA ≥ 3 identified as a significant predictor (OR 3.27), while the mFI-5 showed high sensitivity but low specificity. The association between frailty and postoperative complications was analyzed by Chen et al. [29]. In 515 patients, frail individuals had significantly higher rates of complications, including pulmonary infection (85.1% vs. 26.6%) and arrhythmia (61.9% vs. 9.9%), and mFI-11 demonstrated good predictive performance (AUROC up to 0.832). The longitudinal impact of frailty on recovery was investigated by Chen et al. [30]. Preoperative frailty was present in 28.2% of patients and was associated with significantly worse postoperative quality of life, including impaired physical, role, and social functioning. The predictive utility of frailty and comorbidity indices was compared by Yan [32]. Among 359 patients, major complications occurred in 10.3% of cases, and while the Charlson Comorbidity Index remained a significant predictor (OR 1.52), the mFI-5 was not significantly associated with postoperative outcomes.

## 4. Discussion

Frailty is defined as the progressive decline in the function of multiple organ systems, resulting in a diminished physiological reserve and a reduced ability to withstand stressors, such as the complex demands of cancer surgery [28,37]. Early identification and timely intervention are critical in mitigating the adverse effects of frailty. In the absence of specific laboratory markers available for clinical use, assessment scales have become the primary tools for identifying frailty. These scales assess multiple domains, including physiological, psychological, social, and environmental factors. Commonly used tools include the Fried Frailty Phenotype, frailty index, and FRAIL scale [38,39,40]. Unlike general preoperative risk assessment instruments, frailty-specific scales are tailored to address the unique vulnerabilities and disease characteristics of elderly patients [41]. Numerous studies have demonstrated how frailty increases the risk of serious adverse events and mortality [41,42,43].

This meta-analysis demonstrates that frailty significantly impacts mortality in patients undergoing esophagectomy for esophageal cancer. Frail patients had a markedly increased risk of mortality, with a pooled odds ratio of 4.03. This indicates that frailty is an important determinant of adverse outcomes. This association underscores the significance of incorporating frailty assessments into preoperative evaluations for patients scheduled for esophagectomy. Identifying frail individuals may facilitate more personalized care, including rehabilitation, enhanced perioperative monitoring, and tailored postoperative interventions aimed at mitigating the increased risks of mortality in this patient group. It is crucial to extend clinical assessments beyond general frailty scales by incorporating tools designed to evaluate the nutritional status of patients, such as the Mini Nutritional Assessment (MNA) and its short form (MNA-SF). These nutritional assessment scales offer significant utility in clinical settings, as they not only assess nutritional health but also address other geriatric issues, including mobility, depression, and cognitive decline [44]. Recent evidence suggests that frailty screening should be integrated into standard preoperative protocols to improve patient stratification and optimize perioperative care. Our findings are consistent with recent evidence showing that the prevalence of preoperative frailty in patients with esophageal cancer is high, reaching nearly 30% [6]. Moreover, preoperative frailty has been associated not only with increased postoperative mortality but also with a higher risk of complications and 30-day readmission, further emphasizing its clinical relevance [6]. Together, these data reinforce the need for systematic frailty screening as part of standard preoperative assessment in this patient population. Importantly, recent evidence further strengthens the role of preoperative frailty as a predictor of adverse postoperative outcomes in elderly patients undergoing radical esophagectomy. Hao et al. demonstrated that frailty assessed using the modified 5-item frailty index (mFI-5) was independently associated with significantly increased risks of 30-day mortality (aOR = 14.30), postoperative delirium, and pneumonia [26]. These findings highlight that frailty is not only a general vulnerability marker but also a strong independent predictor of short-term surgical outcomes in this specific patient population. Moreover, the predictive performance of mFI-5 was further improved when combined with age and ASA classification, suggesting that frailty assessment should be integrated with traditional risk stratification tools in preoperative evaluation [26]. Earlier large database analyses have also confirmed the strong association between frailty and postoperative morbidity and mortality following esophagectomy. In a study based on the National Surgical Quality Improvement Program, increasing modified frailty index scores were associated with a stepwise rise in postoperative complications and mortality, with mortality rates increasing from 1.8% in non-frail patients to over 23% in highly frail individuals [25]. Importantly, frailty remained an independent predictor of mortality even after adjustment for other clinical variables, underscoring its prognostic significance. These findings further validate the utility of frailty indices as robust tools for perioperative risk assessment. In addition to commonly used frailty indices such as mFI-5, alternative tools like the Hospital Frailty Risk Score (HFRS) have also been investigated in patients with oesophago-gastric cancer. The HFRS, derived from routinely collected administrative data, enables large-scale frailty stratification and has been shown to correlate with key postoperative outcomes, including prolonged hospital stay, increased 30-day readmission rates, and higher mortality [45]. Notably, studies have demonstrated that the majority of elderly patients undergoing oesophagectomy exhibit some degree of frailty when assessed using HFRS, and outcomes worsen progressively with increasing frailty severity. The use of such scalable tools may facilitate earlier identification of high-risk patients and support implementation of frailty-adapted care pathways in clinical practice.

Importantly, frailty was also associated with a significantly higher risk of selected postoperative complications, particularly those of a cardiopulmonary nature. In our analysis, frail patients had higher odds of postoperative pneumonia (OR 1.86) and cardiac complications (OR 1.73). However, the impact of frailty on other postoperative complications remains an area requiring further investigation. While our analysis did not show statistically significant associations between frailty and surgical site infections or postoperative leakage, the pooled odds ratios suggested a possible increase in the risk of surgical site infections in frail patients, whereas the risk of postoperative leakage appeared lower. The lack of statistical significance may reflect a true absence of association, but it may also be attributed to heterogeneity among studies, small sample sizes in individual cohorts, or variations in perioperative management across centers. These findings highlight the need for larger-scale prospective studies to further clarify the relationship between frailty and specific postoperative complications.

Beyond its impact on mortality and complications, frailty has also been shown to significantly affect postoperative health-related quality of life (HRQoL) in elderly patients undergoing esophagectomy. A prospective longitudinal study demonstrated that preoperatively frail patients experienced significantly worse global quality of life, as well as poorer physical, social, and role functioning compared to non-frail individuals [30]. Furthermore, frail patients exhibited slower postoperative recovery and a persistently higher symptom burden, including fatigue, pain, and sleep disturbances. These findings suggest that frailty influences not only objective surgical outcomes but also patient-reported outcomes, which are increasingly recognized as critical endpoints in oncologic care. Therefore, preoperative frailty assessment may help identify patients at risk of poor postoperative quality of life and guide supportive interventions.

Recent studies suggest that implementing structured prehabilitation programs that include physical exercise, nutritional optimization, and psychological support may enhance surgical outcomes in frail patients. Prehabilitation, defined as improving a patient’s functional capacity before surgery, has shown promise in reducing postoperative complications and hospital length of stay. A recent systematic review and meta-analysis demonstrated that multimodal prehabilitation significantly reduced pulmonary complications and improved recovery in frail esophageal cancer patients [46]. However, further high-quality randomized controlled trials are needed to confirm its efficacy and establish standardized protocols tailored to the esophageal cancer population.

Chronic systemic inflammation has been implicated in the pathogenesis of frailty, and several biomarkers have been identified as potential predictors of postoperative outcomes in frail patients [47,48,49]. Elevated preoperative levels of inflammatory markers such as C-reactive protein (CRP) and neutrophil-to-lymphocyte ratio (NLR) have been associated with increased surgical risks in oncology patients [50,51]. Additionally, emerging biomarkers such as interleukin-6 (IL-6) and fibrinogen have been proposed as indicators of frailty-related complications in major cancer surgeries [52,53,54]. Incorporating inflammatory biomarkers into preoperative risk assessment tools may improve the precision of frailty identification and help tailor perioperative management strategies accordingly.

Neoadjuvant chemotherapy (NAC) is a systemic treatment administered before surgery to achieve tumor downstaging and improve survival and constitutes a standard approach in clinically advanced esophageal cancer. However, its role in elderly and potentially frail patients remains controversial. Yamashita et al. found that in patients aged ≥ 75 years, NAC did not increase postoperative complications overall, but its survival benefit was strongly dependent on baseline performance status [55]. Specifically, patients with a performance status (PS) of 0 derived a significant prognostic benefit from NAC, whereas those with impaired PS (≥1) did not, suggesting that reduced physiological reserve may attenuate the oncologic advantage of preoperative therapy [55]. These findings indicate that frailty-related functional impairment should be carefully considered when balancing the risks and potential benefits of NAC prior to esophagectomy.

A multidisciplinary approach is essential for optimizing perioperative care in frail esophageal cancer patients. Surgeons play a central role in selecting appropriate surgical strategies, while anesthesiologists contribute by optimizing fluid management and analgesia to reduce perioperative stress. Nutritionists and rehabilitation specialists are critical in addressing malnutrition and physical deconditioning, both of which are common among frail patients. Unfortunately, psychological support remains an often overlooked component in the management of frailty. Implementing psychological assessments and interventions, such as structured counseling and cognitive training, may help improve overall patient resilience and compliance with perioperative care.

Although the surgical approach is an important determinant of perioperative risk in esophagectomy, the included studies did not provide sufficiently detailed or consistent data regarding whether procedures were performed via open, minimally invasive (MIE), or robotic-assisted techniques. This represents a relevant limitation, as recent evidence suggests that MIE and robotic esophagectomy may reduce postoperative morbidity and shorten hospital stay [56,57], and, with that, likely also reduce the physiological stress response compared with conventional open surgery, factors that may be particularly meaningful in frail patients. Because most studies enrolled in this meta-analysis were published within the last several years, it is likely that minimally invasive techniques were increasingly used. However, the lack of stratified reporting makes it impossible to evaluate how the surgical approach may have modified the association between frailty and outcomes. Future studies should therefore analyze frailty in the context of specific surgical techniques, as frail patients might derive significantly greater benefit from minimally invasive or robotic approaches than from traditional open approaches.

The present study is subject to certain limitations. Potential biases may arise from the accuracy of data extracted from various publications, which constrains the results of this meta-analysis. Additionally, the authors were unable to conduct certain analyses due to an insufficient quantity of consistent data. Moreover, the analysis was based on cohort studies, and randomized controlled trials are necessary to validate these findings. Furthermore, as surgical techniques, patient characteristics, and perioperative care may differ between Asian and Western populations, this limits the generalizability of our findings. Several factors may account for this discrepancy, including differences in baseline patient comorbidities, obesity prevalence, perioperative infection prevention protocols, and definitions or grading of infections across healthcare systems. Furthermore, variations in surgical approach, case-mix severity, and thresholds for surgical eligibility in Western versus Asian centers may have influenced the observed association between frailty and postoperative infectious complications. It should be noted that in Japan, chemoradiation is available as an alternative to surgery for squamous cell carcinoma, which may bias the selection of patients included in the submitted studies and, consequently, limit the generalizability of our results. Furthermore, none of the included studies reported implementing targeted or intensified preoperative management specifically for frail patients, such as structured prehabilitation, tailored nutritional optimization, or geriatric co-management. Since all cohorts followed standard institutional protocols regardless of frailty status, the potential influence of differential preoperative treatment on postoperative outcomes is likely minimal, but it cannot be fully excluded. Lastly, a formal assessment of publication bias was not performed due to the limited number of studies included in the analyses; this should be considered a limitation of the present study and highlights the need for cautious interpretation of the results. Despite these limitations, this meta-analysis provides valuable evidence on the impact of frailty syndrome in esophageal cancer surgery, supporting the need for targeted perioperative strategies.

## 5. Conclusions

Frailty is a powerful predictor of mortality in patients undergoing esophagectomy, with frail individuals facing nearly four times higher odds of death. This underscores the urgent need to integrate frailty assessments into standard preoperative screening to enhance risk stratification and optimize perioperative decision-making. Although no statistically significant association was found between frailty and certain complications, such as surgical site infections or anastomotic leakage, frailty was significantly associated with an increased risk of postoperative pneumonia and cardiac complications, indicating an overall elevated risk of adverse outcomes. Given these findings, current prehabilitation—including physical training, nutritional optimization, and psychological support—should be a cornerstone of perioperative care for this high-risk population. Additionally, incorporating inflammatory biomarkers into frailty assessment may further refine risk prediction and guide targeted interventions. A multidisciplinary approach is essential to improving resilience, recovery, and long-term survival in frail esophageal cancer patients. Future large-scale prospective trials should focus on standardizing assessment tools and evaluating the lasting impact of tailored interventions to ultimately enhance patient outcomes.

## Figures and Tables

**Table 1:** Characteristics of the studies submitted for this meta-analysis.

First Author	Year	Continent	Country	Total N
Hao, Xinyu [26]	2025	Asia	China	699
Chaochankit, Wongsakorn [27]	2025	Asia	Thailand	127
Boerner, Thomas [28]	2024	North America	USA	447
Chen, Xiu [29]	2024	Asia	China	515
Chen, Xi [30]	2024	Asia	China	131
Shahrestani, Shane [1]	2023	North America	USA	570
Miyauchi, Wataru [22]	2023	Asia	Japan	162
Morito, Atsushi [23]	2023	Asia	Japan	561
Takahashi, Keita [24]	2023	Asia	Japan	239
Yamashita, Kotaro [31]	2023	Asia	Japan	217
Yan, Rui [32]	2023	Asia	China	359
Lee, David [7]	2022	North America	USA	2662
Tanaka, Tsuyoshi [33]	2022	Asia	Japan	69
Park, Mina [34]	2022	North America	USA	45,362
Hodari, Arielle [25]	2013	North America	USA	2095

**Table 2:** Results of the risk-of-bias assessment using the Newcastle–Ottawa scale.

Category	Representativeness of the Exposed Cohort	Selection of the Non-Exposed Cohort	Ascertainment of Exposure	Demonstration That Outcome of Interest Was Not Present at Start of Study	Most Important Factor	Any Additional Factor	Assessment of Outcome	Was Follow-Up Long Enough for Outcomes to Occur	Adequacy of Follow-Up of Cohorts	Total	Quality	Risk of Bias
Hao, Xinyu 2025 [26]	✰	✰	✰	✰	✰	✰	✰	✰	✰	9/9	High	Low
Chaochankit, Wongsakorn 2025 [27]	✰	✰	✰	✰	✰	✰	✰	0	✰	8/9	High	Low
Boerner, Thomas 2024 [28]	✰	✰	✰	✰	✰	✰	✰	✰	✰	9/9	High	Low
Chen, Xiu 2024 [29]	✰	✰	✰	✰	✰	✰	✰	0	✰	8/9	High	Low
Chen, Xi 2024 [30]	✰	✰	✰	✰	✰	✰	✰	0	✰	8/9	High	Low
Shahrestani, Shane 2023 [1]	✰	✰	✰	✰	✰	✰	✰	0	✰	8/9	High	Low
Miyauchi, Wataru 2023 [22]	✰	✰	✰	0	✰	✰	✰	✰	0	7/9	High	Low
Morito, Atsushi 2023 [23]	✰	✰	✰	0	✰	✰	✰	✰	✰	8/9	High	Low
Takahashi, Keita 2023 [24]	✰	✰	✰	0	✰	✰	✰	✰	✰	8/9	High	Low
Yamashita, Kotaro 2023 [31]	✰	✰	✰	✰	✰	✰	✰	✰	✰	9/9	High	Low
Yan, Rui 2023 [32]	✰	✰	✰	✰	✰	✰	✰	0	✰	8/9	High	Low
Lee, David 2022 [7]	✰	✰	✰	✰	✰	✰	✰	✰	✰	9/9	High	Low
Tanaka, Tsuyoshi 2022 [33]	✰	✰	✰	0	✰	✰	✰	✰	✰	8/9	High	Low
Park, Mina 2022 [34]	✰	✰	✰	✰	✰	✰	✰	0	✰	8/9	High	Low
Hodari, Arielle 2013 [25]	✰	✰	✰	0	✰	0	✰	✰	0	6/9	Moderate	Moderate

**Table 3:** Statistical results of this meta-analysis regarding the influence of frailty syndrome on the surgery-related clinical outcomes in patients undergoing esophagectomy.

Category	Odds Ratio	Lower Limit	Upper Limit	Z-Value	*p*-Value	I^2^
Mortality	4.03	2.20	7.38	4.51	0.00	87%
Surgical Site Infection	1.74	0.77	3.94	1.34	0.18	88%
Postoperative Leakage	0.64	0.18	2.28	−0.69	0.49	76%
Postoperative Pneumonia	1.86	1.16	2.98	2.60	0.01	67%
Cardiac Complications	1.73	1.54	1.94	9.37	0.00	-

## Data Availability

The data that support the findings of this study are available from the corresponding author upon reasonable request.

## Supplementary Materials

The following supporting information can be downloaded at: https://www.mdpi.com/article/10.3390/jcm15083040/s1, PRISMA Checklist.
